# The Benefits of Working Memory Capacity on Attentional Control under Pressure

**DOI:** 10.3389/fpsyg.2017.01105

**Published:** 2017-07-10

**Authors:** Xiaoxiao Luo, Liwei Zhang, Jin Wang

**Affiliations:** ^1^Department of Sport Science, Beijing Sport UniversityBeijing, China; ^2^Department of Health Promotion and Physical Education, WellStar College of Health and Human Services, Kennesaw State University, KennesawGA, United States

**Keywords:** attentional control, working memory capacity, state anxiety, working memory training, antisaccade

## Abstract

The present study aimed to examine the effects of working memory capacity (WMC) and state anxiety (SA) on attentional control. WMC was manipulated by (a) dividing participants into low- and high-WMC groups (Experiment 1), and (b) using working memory training to improve WMC (Experiment 2). SA was manipulated by creating low- and high-SA conditions. Attentional control was evaluated by using antisaccade task. Results demonstrated that (a) higher WMC indicated better attentional control (Experiments 1 and 2); (b) the effects of SA on attentional control were inconsistent because SA impaired attentional control in Experiment 1, but favored attentional control in Experiment 2; and (c) the interaction of SA and WMC was not significant (Experiments 1 and 2). This study directly manipulated WMC by working memory training, which provided more reliable evidence for controlled attention view of WMC and new supportive evidence for working memory training (i.e., far transfer effect on attentional control). And the refinement of the relationship between anxiety and attentional control proposed by Attentional Control Theory was also discussed.

## Introduction

Attentional control is one of the key components of human perception, which requires an individual to focus on the task-relevant information and resist the interference of task-irrelevant information (i.e., distractor) (Knudsen, 2007). It is important for people who would like to maintain concentrated for certain task, especially under some stressful situations that may induce anxiety, such as examination, surgery, aviation, and competitive sports.

The controlled attention view of working memory capacity (WMC; Kane et al., 2001; Engle, 2002) suggests that WMC is not about individual differences in how many items can be stored per se but about differences in the ability to control attention to suppress interference, avoid distraction and maintain information in an active, quickly retrievable state. More important, WMC and attentional control share a similar neural system (i.e., prefrontal cortex) (Kane and Engle, 2002; van Veen and Carter, 2006). The controlled attention view of WMC proposed that high-WMC individuals are generally better able to maintain top–down attentional control and remain focused, whereas low-WMC individuals are likely to experience failures in goal maintenance due to their inability to inhibit distraction or interference (Kane et al., 2001; Engle, 2002; Barrett et al., 2004; Unsworth et al., 2004). There are numerous studies using various paradigms support this prediction (e.g., Kane et al., 2001; Unsworth et al., 2004; Colflesh and Conway, 2007; Fukuda and Vogel, 2011; Furley and Memmert, 2012; Hiebel and Zimmer, 2015).

Another separate line concerning attentional control is about anxiety and attentional control. Attentional Control Theory (Eysenck et al., 2007) proposed that anxiety creates an imbalance between two attentional systems: goal-directed (top–down) system (responsible for the maintenance of task goals) and stimulus-driven (bottom–up) system (sensitive and responsive to salient stimuli). Successful attentional control requires the processing of goal-directed system, whereas anxiety will decrease the influence of goal-directed system and increase the influence of the stimulus-driven system. In other words, anxiety will impair attentional control and lead to distraction. This prediction was supported by many researchers (e.g., Derakshan et al., 2009; Wilson et al., 2009a; Edwards E.J. et al., 2015; for reviews see Derakshan and Eysenck, 2009; Eysenck and Derakshan, 2011), especially sport psychologists (e.g., Wilson et al., 2009b; Wood and Wilson, 2010; Nieuwenhuys and Oudejans, 2010; Causer et al., 2011; Navarro et al., 2013).

One important issue in Attentional Control Theory is the type of anxiety. Anxiety can be differentiated into trait anxiety (TA) and state anxiety (SA) (Spielberger et al., 1983). TA is a personality dimension characterized by a stable and chronic propensity to experience moderate to high levels of anxiety in general, whereas SA is a more acute and transient emotional experience of anxiety triggered by situational stress or pressure. Previous studies about Attentional Control Theory provided consistent evidences for the prediction that TA will impair attentional control (e.g., Ansari et al., 2008; Derakshan et al., 2009; Moser et al., 2012; Edwards E.J. et al., 2015). However, there were inconsistent evidences for the effect of SA on attentional control, which challenged Attentional Control Theory (e.g., SA showed negative effect: Wood and Wilson, 2010; Navarro et al., 2013; Allsop and Gray, 2014; SA showed null effect: Moser et al., 2012; Edwards E.J. et al., 2015; Hoskin et al., 2015; SA showed positive effect: Booth and Sharma, 2009). It is worthy to further explore the effect of SA on attentional control. We think that the inconsistent effects of SA could attribute to the different SA levels in different studies, because the SA conditions are different in different studies so that the induced SA levels are different. Relationship between SA levels and task performance is supposed as an inverted-U curve, and a moderate SA will favor task performance (Yerkes and Dodson, 1908; Spielberger et al., 1983; Jones, 1995). Although task performance is not equal with attentional control, we think that the effect of SA on task performance could prompt the effect of SA on attentional control. We infer that there could be two different effects of SA on attentional control (i.e., both negative and positive effects, which conflict with each other). Considering that the effect of SA is unclear in contrast to TA as discussed above, the present study would focus on SA.

Given the views of Attentional Control Theory (i.e., anxiety impairs attentional control) and controlled attention view of WMC (i.e., high-WMC favors attentional control), it is very possible that WMC could modulate the effect of anxiety on attentional control, that is, high-WMC individuals with high-TA will be better at attentional control compared with low-WMC individuals with high-TA; or high-WMC individuals will be better at attentional control under pressure (i.e., under SA condition) compared with low-WMC individuals. To date, several studies have explored the effects of WMC and anxiety (including TA and SA) on attentional control (Booth and Sharma, 2009; Johnson and Gronlund, 2009; Edwards M.S. et al., 2015; Wood et al., 2015; Wright et al., 2014), but the results of these studies are inconsistent. For example, two studies (Johnson and Gronlund, 2009; Wright et al., 2014) found significant interaction of WMC and anxiety (i.e., the deficit of attentional control under anxiety was less obvious for high-WMC individuals compared with low-WMC individuals, and it should be mentioned that the “anxiety” in these two studies was TA), but Wood et al. (2015) did not find this interaction (the “anxiety” in this study was SA). Furthermore, Booth and Sharma (2009) even found a significant interaction with opposite pattern (i.e., the increase, not deficit, of attentional control under anxiety was more obvious for high-WMC individuals compared with low-WMC individuals, and the “anxiety” here was SA).

There might be two reasons for obtaining these different results. First, the manipulations of anxiety are different. Studies employed TA showed consistent results, whereas studies employed SA showed inconsistent results. This pattern is similar with studies on Attentional Control Theory mentioned above. So we did a similar inference that different SA conditions induce different SA levels so that the effects of SA were inconsistent. For example, Edwards M.S. et al. (2015) might have induced relatively low SA level, because they did not manipulate SA conditions directly (they only measured the SA levels using questionnaires after experiment); Booth and Sharma (2009) might have induced relatively moderate SA level, because they used single-source SA condition: noise punishment (that is why the SA effect in this study was positive, because moderate SA might favor performance); Wood et al. (2015) might have induced relatively high SA levels, because they used multi-sources SA condition: gun shooting threat and peer comparison. So we argue that multi-sources SA condition could be a better way to induce SA, and we would also employ this SA condition in the present study.

Second, some attentional control measurements in these studies (e.g., Stroop task, Booth and Sharma, 2009; highly demanding dual-task, Johnson and Gronlund, 2009; attentional shifting task, Edwards M.S. et al., 2015) confounded attentional control (e.g., the eye movement data) and task performance (e.g., accuracy or reaction time), which might make the indicators less sensitive. Attentional Control Theory claims that anxiety impairs attentional control but did not affect task performance directly (Eysenck et al., 2007), because one can invest more efforts to maintain good performance when attentional control is impaired. So a better attentional control measurement is eye movement (Wright et al., 2014; Wood et al., 2015), because the fixation often cued the focus of attention. For example, antisaccade task is often used in studies on Attentional Control Theory (e.g., Derakshan et al., 2009; Wright et al., 2014), and we would also employ this task in the present study.

Another problem with studies concerning the interaction of anxiety and WMC on attentional control is that no study manipulates WMC directly. A regular approach to explore the effect of WMC is to divide participants into low-WMC group and high-WMC group based on performance of WMC tasks (operation-word span task, i.e., OSPAN is one of the most commonly used measurements). One could argue that the causal link between WMC and attentional control is not solid due to the lack of experimental manipulation. Here we could manipulate WMC directly by using working memory training (WM training). The plasticity of WMC was widely explored in the past decade, numerous studies indicated that WM training could improve WMC, which is regarded as “near transfer” (e.g., Klingberg et al., 2002; Dahlin et al., 2008; Chein and Morrison, 2010; Jaeggi et al., 2011; for review see Shipstead et al., 2012). And the benefits of training could transfer to other aspects such as fluid intelligence (see Au et al., 2015 for meta analyses) or attentional control (e.g., Klingberg et al., 2005; Brehmer et al., 2011; Borella et al., 2014; see Karbach and Verhaeghen, 2014, for meta analyses on older adults), which is regarded as “far transfer.” It should be mentioned that the effectiveness of far transfer is still unclear (some researchers did not support far transfer effect such as Shipstead et al., 2012; Melby-Lervåg and Hulme, 2013; Melby-Lervåg et al., 2016, and the previous studies about far transfer effect on attentional control also produce inconsistent evidences), but the effectiveness of near transfer is widely supported. So we could use WM training to manipulate WMC directly to further confirm the causal link between WMC and attentional control and, at the same time, examine the far transfer (i.e., attentional control) of WM training.

In the present study, we conducted two experiments to examine the effect of WMC and SA on attentional control. Considering the existing problems of studies on this topic mentioned above, multi-sources SA conditions were used to induce relatively high-SA level (Experiments 1 and 2), and antisaccade task was used to evaluate attentional control for a more direct attentional control measurement and separating attentional control from task performance (Experiments 1 and 2). OSPAN was used to evaluate WMC, we divided participants into low- and high-WMC groups based on original OSPAN scores (Experiment 1), and we also manipulated WMC directly using WM training (adaptive n-back training) (Experiment 2). Results of Experiment 1 showed that SA impairs attentional control and high-WMC individuals were better at attentional control, but the interaction of SA and WMC was not significant. Results of Experiment 2 showed that individuals with WM training (i.e., WMC had improved) were better at attentional control compared with individuals without WM training (i.e., WMC had not improved).

## Experiment 1

Previous studies showed inconsistent results of the effects of WMC and anxiety on attentional control as mentioned in Introduction. Here we conducted Experiment 1 to examine this effect again and attempted to solve potential problems in previous studies as mentioned above. Antisaccade task was used to evaluate attentional control. We divided participants into low- and high-WMC groups based on OSPAN scores and manipulated low- and high-SA using multi-sources SA condition. One reason for choosing SA rather than TA as an anxiety independent valuable is that SA could be manipulated in experiment by setting stressful situation. Nevertheless, we also measured TA as a covariant variable, because previous studies provided consistent evidences for TA impairs attentional control as mentioned in Introduction (e.g., Ansari et al., 2008; Derakshan et al., 2009; Moser et al., 2012; Edwards E.J. et al., 2015). The present study, however, focused on SA (that is, we did not concern about the effect of TA, but there still might be individual differences on TA), so we considered to regard TA as covariant variable to balance the contribution of TA on attentional control. There were three hypotheses in Experiment 1:

H1-1: SA impairs attentional control, that is, the first correct antisaccade latency (latency) would be longer and the percentage of incorrect saccades (error rate) would be higher under high-SA condition compared with low-SA condition. This hypothesis aimed to explain previous inconsistent results of SA on attentional control.

H1-2: high-WMC individuals have better attentional control, that is, the latency would be shorter and the error rate would be lower for high-WMC group compared with low-WMC group. This hypothesis is a replication of previous studies.

H1-3: WMC modulates the effect of SA on attentional control, that is, the increase of latency and error rate under high-SA would be less obvious for high-WMC group compared with low-WMC group. This hypothesis aimed to explain previous inconsistent results of interaction between SA and WMC.

### Method

#### Participants

Sixty four healthy young adults were recruited by flyer. All participants provided informed consent in advance and received ¥50 payment for their participation. This experiment was approved by Beijing Sport University Institutional Review Board (BSUIRB) (Approval Number: 2015037). However, one participant claimed that SA condition was not effective for him/her (he/she felt that he/she was more anxious under low-SA condition compared with high-SA condition. More important, the data of his/her manipulation check also indicated that he/she was strangely far more anxious under low-SA condition compared with high-SA condition) so this data were excluded. Furthermore, seven participants who performed over 40% invalid trials were also excluded (according to Derakshan et al., 2009, for the detail of exclusion criteria, see “measurement of attentional control” below). After the data collection, 56 participants were included (13 males, 43 females; mean age 21.339 ± 2.414 years. In terms of the education level, there were 40 undergraduate students and 16 graduate students in these 56 participants).

#### Measurement of WMC

Operation-word span task (La Pointe and Engle, 1990) was used to evaluate WMC, which has been widely approved (e.g., Engle et al., 1999; Kane et al., 2001; Unsworth et al., 2004; Wright et al., 2014; Wood et al., 2015). In OSPAN, an operation-word string [e.g., (3 × 3)-5 = 4? Train] will be displayed on the screen and participant is required to read the operation aloud, verify aloud whether the operation is correct (“right” vs. “false”; 85% accuracy criterion on the operations is required for all participants), and then finally read the word aloud. Once the participant has read the word aloud, the experimenter presses a key to move onto the next operation-word string. Pausing was not permitted during this process until three question masks (???) cued the participant to recall the words from that set in the correct order (write the words on an answer sheet). The operation-word strings can vary from two to six items in length and each length has three sets (the different set sizes appear in an unpredictable order). Thus, the OSPAN score is the sum of the recalled words for all sets recalled completely in correct order (if participant has recalled words completely but in wrong order, half of the words in this set will be included in the score), and all possible scores that ranged from 0 to 60. Higher OSPAN scores imply higher WMC. This OSPAN task program was developed using E-prime 2.0.

#### Measurement of Attentional Control

**FIGURE 1:**
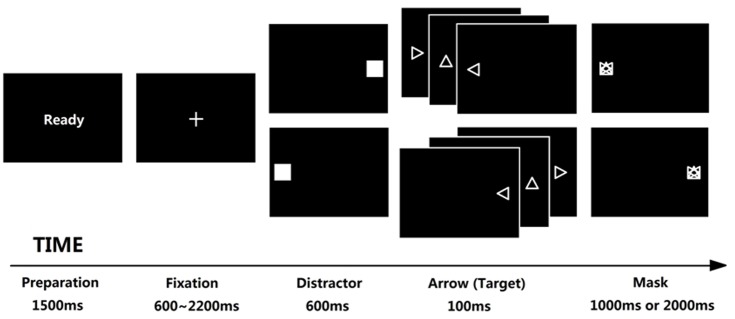
Trial structure of antisaccade task. Each trial begins with “Ready” reminding participant to prepare; the fixation was displayed for 600 ∼ 2200 ms randomly in order to avoid anticipatory saccade, any saccades before the fixation disappears would be regarded as invalid trials; a flashing square appears for 600 ms either left or right immediately after the fixation disappears, and each participant was required to direct their gaze away from it as quickly as possible (the lower latency of this saccade implies the higher ability of attentional control). Then an additional arrow appears at the opposite location of flashing square for 100 ms, requiring each participant to judge the direction of the arrow (left, right, or up) before the mask disappears; the mask would be displayed for 1000 or 2000 ms under low- or high-SA condition, respectively; if a participant made a wrong response or did not respond in the limited time, this trial would be regarded as a wrong trial, leading to a noise punishment for 500 ms under high-SA condition (which did not show in this figure).

Tobii T120 was used as the eye-tracking device with 120 Hz sampling rate. The stimuli was displayed on the Tobii Eye Tracker (subtending 32.47° × 25.79°, resolution is 1024 × 768 pixels, and refresh rate is 60 Hz). The distance between participant’s eyes and the center of screen was 60 cm. Antisaccade task (Hallett, 1978) was used to evaluate attentional control (Kane et al., 2001; Unsworth et al., 2004; Hutton and Ettinger, 2006; Derakshan et al., 2009; Wright et al., 2014), which was developed using E-prime 2.0. In this task, attentional control was required to suppress a reflexive saccade toward a distractor, and generate a volitional saccade to its mirror position (see **Figure 1** for trial structure). Each trial begins with “Ready” in the center of screen for 1500 ms and participant is required to fixate a cross (1.2 cm × 1.2 cm, subtending 1.15° × 1.15°, displaying for random 600 ∼ 2200 ms) until it disappears. A flashing square (i.e., distractor, 5.5 cm × 5.5 cm, subtending 5.25° × 5.25°, displaying for 600 ms) then appears either left or right of the center at 13.37° with equal possibility. The participant is then required to direct their gaze AWAY from the flashing square in the opposite location as quickly as possible. Immediately after the presentation of square, a triangle arrow (1 cm × 1 cm, subtending 0.96° × 0.96°, displaying for 100 ms) appears at 13.37° from the center in the opposite direction of the square and followed with a mask (1 cm × 1 cm, subtending 0.96° × 0.96°, displaying for 1000 or 2000 ms in high- and low-SA condition, respectively). The participant needs to identify the arrow’s direction (up, left or right) within limited time by pressing the relevant keys on the keyboard. In fact, we don’t concern the arrow-judgment because it is an explicit task requirement represented for task performance, not the indicator of attentional control (we concern attentional control rather than task performance). In contrast, the eye movement data in antisaccade task are rather implicit indicators, which are suitable as indicators of attentional control (as mentioned in Introduction, the advantage to use antisaccade task is that we could separate attentional control (AC) from task performance). At last, we only analyzed the eye movement data and ignored the task performance data. Nevertheless, the requirement to identify the arrow direction ensured participants were more engaged in the task.

The two main dependent variables (eye movement data) were the latency of first correct saccade (latency, being the elapsed time between the onset of the distractor and a saccade in the correct direction before the onset of arrow, which reflects the effort of suppressing the attraction of distractor and implies the deficit of attentional control if the participant needed more time to complete a correct saccade) and the percentage of incorrect saccades (error rate, being the percentage of the trials that saccade in the distractor’s direction, which reflects the trends of being attracted by distractor and also implies the deficit of attentional control if participant performed more incorrect saccades). According to Derakshan et al. (2009), a first correct saccade was defined as a first eye movement with a velocity > 30°/s and amplitude > 3° toward the mirror position of distractor that was made after the onset of the distractor and before the onset of arrow. Similarly, an incorrect saccade was the first saccade toward the position of the distractor after it onset. Trials that latency shorter than 83 ms (i.e., anticipatory) or longer than 600 ms (i.e., saccade failed) were excluded. Also, trials would be regarded as invalid trial when the eye tracker failed to sample that trial. There were 36 trials in 1 block. Participants’ data would be excluded if they performed more than 15 trials (over 40%) should be excluded (all these exclusion criteria above were according to Derakshan et al., 2009, and 7 data were excluded in Experiment 1).

#### The SA Condition

Multi-sources SA condition (i.e., limited wrong response, limited reaction time, noise punishment, and electric shock threat) were used to induce SA. There were two SA conditions: low- and high-SA. In the low-SA condition, each participant was required to keep the number of wrong judgments of the arrow direction (error times) within three (i.e., maximum error times were three), and the reaction time to judge the arrow direction was limited within 2 s, or this trial would be regarded as a wrong judgment (i.e., maximum RT was 2 s). Whereas in the high-SA condition, the maximum error time was only one and the maximum RT was 1 s. Once a participant made a wrong judgment, he/she would be punished by white noise (lasting 500 ms, which was generated by Cool Edit Pro V2.1, and presented via EDIFIER headphones. The actual intensity of the noise is 95.730 ± 2.545 dB measured by using BENETECH GM1356 decibel device for 10 times. The participant was told the intensity is 110 dB, and would listen to the noise before high-SA condition task in order to ensure the effectiveness of inducing high-SA). Moreover, the experimenter would stand aside and hold an electric stimulator (TAIMENG BL-420S Biological Experimental System with 24V DC power). Participant was told that he/she could be shocked by the electric stimulator at any time (may not immediately) if a wrong judgment was made (but it actually was just a kind of electric threat, because participant would never be shocked during the experiment). Besides, both physiological and psychological measurements were used as SA manipulation check: participant’s heart rate and skin conductance were recorded during the experiment using biofeedback device (NEXUS-10 MARK II) as physiological measurement, and participant was also required to fill the mental readiness form-3 (MRF-3, Krane, 1994) as psychological measurement. MRF-3 is an 11-point Likert scale with three items to evaluate cognitive anxiety, somatic anxiety and state confidence, which is applicable to measure the SA (Krane, 1994; Wilson et al., 2009b; Wood and Wilson, 2010).

#### Procedure

Participants completed the Trait-Anxiety Inventory (T-AI, part of the State-Trait Anxiety Inventory, Spielberger et al., 1983) after informed consent. Then, they completed the OSPAN, including practice (2 sets with 5 operation-word strings) and formal task (15 sets with 60 operation-word strings). After that, participants could have a break and the experimenter would help them equipping the biofeedback (heart rate and skin conductance would be recorded from now on till the end of experiment). Participants subsequently conducted a practice block of antisaccade task (12 trials), and then completed a practice block again after calibration of the eye-tracker. In the formal antisaccade task, participants needed to conduct a low-SA block and a high-SA block, respectively. Each block had 36 trials, and participants were required to complete the MRF-3 after each block (the sequence of low- and high-SA block was determined by random lottery). At last, participants were interviewed briefly.

### Results

We did the manipulation check of SA condition first. The evaluations of SA level (heart rate, skin conductance, and MRF-3 scores) were analyzed. The heart rate, *F*(1,55) = 38.320, *p* < 0.001, $\eta_{p}^{2}$ = 0.411, skin conductance, *F*(1,55) = 22.639, *p* < 0.001, $\eta_{p}^{2}$ = 0.292, and MRF-3 scores, *F*(1,55) = 92.905, *p* < 0.001, $\eta_{p}^{2}$ = 0.628, were all significantly increased in the high-SA condition, implying that SA manipulation was successful.

Then, 56 participants were sorted based on the OSPAN scores, and half of them were selected from the top half of the distribution as high-WMC group (*n* = 28), and another half as low-WMC group (*n* = 28), see **Table 1** for the descriptive data of OSPAN scores. We originally would like to conduct a 2 (SA Condition as within-participants factor: low vs. high) × 2 (WMC Group as between-participants factor: low vs. high) ANCOVA for the two indicators (i.e., latency and error rate, respectively) of attentional control, and the covariant variable was TA measured by T-AI. But the preliminary analysis revealed that TA was not applicable as a covariant variable^1^, so typical 2 × 2 ANOVA was then conducted for latency and error rate respectively, regardless of TA (it should be noted there was no significant difference on T-AI scores between low- and high-WMC group, *t*(54) = -0.439, *p* = 0.662, demonstrating that that TA is not the main contributor of the effects on latency or error rate). See **Table 1** for the descriptive data of latency and error rate.

**Table 1:** Working memory capacity and attentional control in Experiment 1 (means, with standard deviations in parentheses).

Indicators	Low WMC		High WMC	
	Low-SA	High-SA	Low-SA	High-SA
**WMC**				
OSPANs	11.679 (3.418)			22.875 (5.319)	
**Attentional control**				
Latency	391.094 (43.065)	407.443 (44.490)	354.965 (38.808)	370.109 (43.580)
Error rate	0.233 (0.194)	0.261 (0.194)	0.213 (0.155)	0.252 (0.160)

*WMC, working memory capacity; SA, state anxiety; WM training, the working memory training group; Control, the control group; OSPANs, operation-word span task scores; Latency, the latency of first correct saccade; Error rate, the percentage of incorrect saccades*.

**FIGURE 2:**
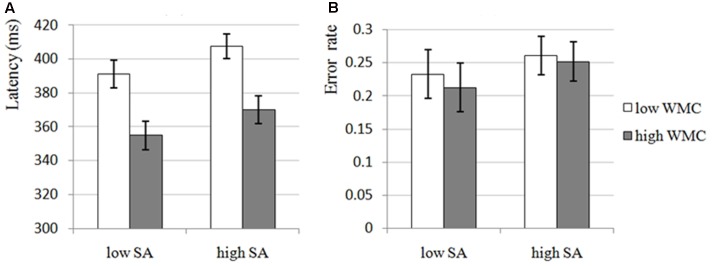
The attentional control of low and high-WMC group under low and high-SA condition in Experiment 1. SA, state anxiety; WMC, working memory capacity. **(A)** Latency, the latency of first correct saccade, which is an indicator of attentional control, reflects the deficit of attentional control if participant need longer latency; in Experiment 1, the main effects of SA and WMC were significant, but the SA × WMC interaction was not significant. **(B)** Error rate, the percentage of incorrect saccades, which is also an indicator of attentional control, reflects the deficit of attentional control if participant performed more incorrect saccades; in Experiment 1, only SA was found to have significant main effect, the main effect of WMC and the SA × WMC interaction were not significant.

The results of 2 × 2 ANOVA for latency and error rate showed that the main effects of SA Condition were significant for both latency, *F*(1,54) = 12.988, *p* = 0.001, $\eta_{p}^{2}$ = 0.194, and error rate, *F*(1,54) = 6.199, *p* = 0.016, $\eta_{p}^{2}$ = 0.103, that is, there were significant increases in high-SA condition compared with low-SA condition for both latency (see **Figure 2A**) and error rate (see **Figure 2B**), which was consistent with H1-1, demonstrating that high-SA impairs attentional control. Furthermore, the main effects of WMC Group were significant for latency, *F*(1,54) = 12.246, *p* = 0.001, $\eta_{p}^{2}$ = 0.185, but not for error rate, *F*(1,54) = 0.103, *p* = 0.749, $\eta_{p}^{2}$ = 0.002, that is, there was a significant decrease in high-WMC group compared with low-WMC group for latency (see **Figure 2A**), but not for error rate (see **Figure 2B**), which was still consistent with H1-2, demonstrating that high-WMC individuals have better attentional control (the non-significant result for error rate would not affect this inference too much, and it would be discussed in the Section “General Discussion”). Unfortunately, none of the interactions were significant (all *ps* > 0.663), which was inconsistent with H1-3, demonstrating that the effects of SA and WMC on attentional control seems to be independent with each other.

### Discussion

In Experiment 1, we manipulated low- and high-SA and divided participants into low- and high-WMC group to examine the effects of SA and WMC on attentional control. Results completely supported Attentional Control Theory (i.e., H1-1): attentional control was impaired under high-SA (see also Wilson et al., 2009a; Wood and Wilson, 2010; Navarro et al., 2013; Allsop and Gray, 2014). Given that we used multi-sources SA condition to induce relatively high-SA and got this result, it implies that the inconsistent results of SA on attentional control might be explained by the different SA levels induced by different SA conditions in different studies. Comparing with TA, the effect of SA is more complex due to the inference that the effect depends on the SA level: the positive effects of SA (e.g., improvement of motivation or arousal) might counteract the negative effects of SA (e.g., impairment of attentional control) if the SA level is relatively low or moderate.

Furthermore, the results of Experiment 1 supported controlled attention view of WMC (i.e., H1-2): high-WMC individuals have better attentional control (see also Conway et al., 2001; Kane et al., 2001; Unsworth et al., 2004; Colflesh and Conway, 2007; Fukuda and Vogel, 2009, 2011; Unsworth and Spillers, 2010; Unsworth and Robison, 2016). High-WMC individuals may get more attentional resources to cope with the distractor (distractor cannot be avoided in many cases) during the time when they were doing the main task. It should be mentioned, however, that this kind of benefit was not observed for error rate (in fact, we would see a similar null-effect again in Experiment 2 and this will be discussed in the Section “General Discussion”).

Unfortunately, the results of Experiment 1 did not support the prediction of the possible interaction (i.e., H1-3): the SA × WMC interaction did not affect attentional control (see also Wright et al., 2014; Wood et al., 2015). Wright et al. (2014) suspected that the mathematical operations in the OSPAN task may be anxiety provoking, leading to an underestimation of WMC. This implies that OSPAN may lead to negative feeling (e.g., low self efficacy) due to higher difficulty than other WMC measurements, which may affect task performance. But OSPAN is one of the most effective measurements of WMC (Engle et al., 1999). So we speculate that the effect of SA and WMC on attentional control might be independent with each other, that is, SA and WMC might affect different aspects of attentional control (this will be discussed in Section “General Discussion”).

## Experiment 2

In Experiment 1, we divided participants into low- and high-WMC group based on OSPAN scores, just like most of previous studies (e.g., Conway et al., 2001; Kane et al., 2001; Unsworth et al., 2004; Colflesh and Conway, 2007; Fukuda and Vogel, 2011; Furley and Memmert, 2012; Hiebel and Zimmer, 2015; Wood et al., 2015). However, one shortcoming of these studies (including Experiment 1) was that researchers did not manipulate WMC directly (they divided participants into low- and high-WMC groups based on original WMC rather than directly manipulate WMC), which relied heavily on samples, and it was not enough to infer the relationship between WMC and attentional control. Experiment 2 was conducted to explore the effect of SA and WMC on attentional control again, and we manipulated both SA (the same with Experiment 1) and WMC (using WM training).

It is reasonable to use WM training to manipulate WMC, because the near transfer effect (i.e., WMC would be improved after WM training) has been widely supported in previous studies (see Melby-Lervåg and Hulme, 2013; Karbach and Verhaeghen, 2014; Au et al., 2015; Melby-Lervåg et al., 2016 for meta analysis). Besides, however, the far transfer effect (i.e., whether the benefits of WM training would transfer to attentional control) is still unclear. Shipstead et al. (2012) suggested that studies in this area have far relied heavily on the Stroop task to evaluate attentional control, future studies should employ a variety of tasks that converge on the attention construct such as antisaccade task. So Experiment 2 could also examine the far transfer effect of WM training using antisaccade task (which is a novel task in this field) to evaluate attentional control. There were three hypotheses in Experiment 2 (it should be mentioned that we did not find the SA × WMC interaction in Experiment 1 and we speculate that the effects of SA and WMC are independent with each other, so we did not propose any prediction about interaction effects in Experiment 2):

H2-1: WMC will be improved after WM training (i.e., near transfer), that is, the OSPAN scores of WM training group would be higher compared with control group after training. This hypothesis is a replication of previous studies.

H2-2: individuals with WM training have better attentional control after training (i.e., far transfer), that is, the latency and error rate of WM training group would be lower compared with control group after training. This hypothesis would provide a more reliable evidence for the causal relationship of WMC and attentional control.

H2-3: SA impairs attentional control, that is, the latency and error rate would be higher under high-SA condition compared with low-SA condition. This hypothesis would be helpful for explain previous inconsistent results of effects of SA on attentional control (taken together with H1-1).

### Method

#### Participants

Thirty two participants of Experiment 1 were selected as the participants of Experiment 2. The screening process was (a) 7 of 56 participants in Experiment 1 were excluded first (they performed more than 10 invalid trials in one antisaccade block) to ensure more acceptable data in Experiment 2; (b) the experimenter invited participants to attend Experiment 2 one by one from the rest of 49 participants based on the order of OSPAN scores in Experiment 1 (from lowest to highest) to ensure more obvious training effect in Experiment 2; (c) at last, we had sent 36 invitations and 32 participants accepted. And the training sessions were started 1 week after Experiment 1 (without training, the WMC should be stable in this kind of short period). Participants provided informed consent in advance and received ¥200 payment for their participation. This experiment was approved by Beijing Sport University Institutional Review Board (BSUIRB) (Approval Number: 2015037). These 32 participants’ data in Experiment 1 were regarded as the pre-training data in Experiment 2, and they were randomly (toss) matched into WM training group (*n* = 16) and control group (*n* = 16) based on OSPAN scores, there was no significant difference on pre-training OSPAN scores between WM training and control group, *t*(30) = -0.156, *p* = 0.877. At last, no data were excluded according to the same criteria in Experiment 1, so all 32 participants were included (9 male, 23 female; mean age 21.000 ± 1.481 years).

#### Training Task

Adaptive spatial n-back training (Jaeggi et al., 2011, 2014) was utilized for the WM training group, and adaptive spatial 1-back training was utilized for the control group. In a spatial n-back task, participants were presented with a sequence of stimuli (i.e., a blue square) appearing at random spatial locations on the screen, one at a time at a rate of 3 s (stimulus length is 500 ms; judgment interval is 2500 ms). Participants were required to press a key whenever the currently presented stimulus was at the same location as the one *n* item(s) back in the series (targets), and press another key if that was not the case (non-targets). There were 7 targets and 14 non-targets of trials per block (which included 21 + n trials). Whereas in a spatial 1-back task, the most important difference with the n-back task above is that participants were required to press a key whenever the currently presented stimulus was at the same location as the previous one in the series, and press another key if that was not the case.

In each training session, participants in both groups were required to complete 15 blocks, which lasted 20∼30 min. For WM training group, the difficulty level was adjusted according to the participants’ performance after each block (see **Table 2**, participants started from level 1. If the error times were less than the requirement, then participants would pass this level and the present level would increase one; if the error times were more than the requirement, then participants would stay in the present level and repeat this level; if participants failed to pass one certain level for three times, than the present level would decrease one). For the control group, the number of n was always 1 (which would not affect WMC in general), and the difficulty level was also adjusted according to participants’ performance. The difficulty level was manipulated by changing the interval of judgment, number of blocks in one level, and the error times allowed in one level (see **Table 2** for details), that is, we designed the tasks for control group like a fast-response game (i.e., the response interval would be shorter and shorter along with the increasing of difficulty level), besides, the blocks that participants should complete would also increase along with the increasing of difficulty level, and the error times allowed in one level would also be changed based on difficulty level. This kind of design could ensure that the treatment of WM training and control group was almost the same (including the improvement of achieved difficulty level based on participants’ performance which is known as “adaptive task”), but the control group was always conducting 1-back task. The last achieved difficulty level would be recorded after participants had finished one training session (i.e., 15 blocks), and then participants would start the next training session from this level.

**Table 2:** The difficulty levels for WM training group and control group.

Level	WM training group					Control group				
	Task	spatial	Block	Time	Error(s)	Task	spatial	Block(s)	Time	Error(s)
1	1-back	7	1	2500	2	1-back	25	1	1000	2
2	1-back	9	1	2500	2	1-back	25	2	1000	4
3	2-back	9	1	2500	2	1-back	25	2	700	4
4	2-back	11	1	2500	2	1-back	25	3	700	6
5	3-back	11	1	2500	2	1-back	25	3	600	6
6	3-back	13	1	2500	2	1-back	25	4	600	8
7	4-back	13	1	2500	2	1-back	25	4	550	8
8	4-back	15	1	2500	2	1-back	25	5	550	10
9	5-back	15	1	2500	2	1-back	25	5	500	10
10	5-back	17	1	2500	2	1-back	25	5	500	5
11	6-back	17	1	2500	1	1-back	25	5	400	5
12	6-back	19	1	2500	1	1-back	25	5	400	1
13	7-back	19	1	2500	1	1-back	25	5	300	5
14	7-back	21	1	2500	1	1-back	25	5	300	1
15	8-back	21	1	2500	1	1-back	25	5	250	5
16	8-back	23	1	2500	1	1-back	25	5	250	1
17	9-back	23	1	2500	1	1-back	25	5	230	5
18	9-back	25	1	2500	1	1-back	25	5	230	1
19	10-back	25	1	2500	1	1-back	25	5	220	5
20	10-back	27	1	2500	1	1-back	25	5	220	1

*Task, the task participants should complete in this level; Spatial, the number of possible locations of the stimuli; Block(s), the number of blocks participants should complete in this level; Time, maximum response time of each trial in this level (ms); Errors, maximum error times allowed to pass this level*.

There were 15 training sessions for both groups, and participants would complete one session per day. After each training session, participants were required to answer 3 manipulation check questions which were (a) how concentrated do you think you were in this training session (i.e., perceived attention level); (b) how difficult do you think the task was in this training session (i.e., perceived difficulty level); and (c) how attractive do you think the task was in this training session (i.e., perceived attraction level). All these questions were 7-points Likert evaluation. Participants could get “points” after they achieved a higher level, and they could get extra monetary reward based on how many points they have at the end of all training sessions.

#### Measurement of WMC, Measurement of Attentional Control, and the SA Condition

All were the same with Experiment 1.

#### Procedure

Participants were invited to take part in Experiment 2 and matched into WM training group and control group based on pre-training OSPAN scores (i.e., OSPAN scores measured in Experiment 1). Both groups started training at the same time, and participants were required to go to the lab every day to complete one training session within a certain period of time. If someone could not go to the lab due to any problem, the experimenter would send participants an Email with the training program attached. Participants would then complete the training session whenever convenient, and send the training data back to experimenter (This occurred 1 time for 8 participants, 2 times for 1 participant, and 3 times for 3 participants in the total 15 times of training). If someone even did not have time to complete one training session 1 day, there would be a break and participants needed to be trained one more day to achieve the required number of training sessions (This occurred 1 time for 1 participant, and 2 times for 2 participants in the total 15 times of training). After 15 training sessions, participants conducted OSPAN and antisaccade task like Experiment 1 (also under same SA conditions as in Experiment 1). At last, participants were interviewed briefly.

### Results

#### Profile of WM Training

**FIGURE 3:**
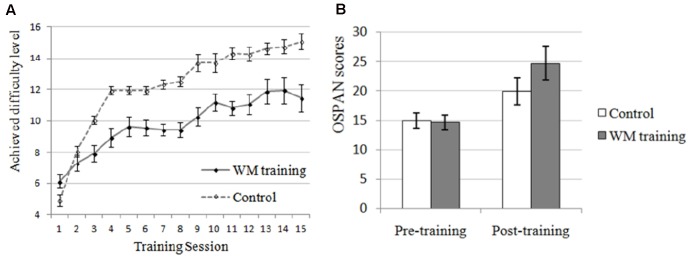
Effects of working memory (WM) training on trained and untrained task in Experiment 2. **(A)** WM training group performed better and better on the training task (n-back) along with the increase of training sessions, demonstrating that the WMC of participants in WM training group was improved (they completed n-back task, and the number of *n* was increased along with the enhancement of achieved difficulty level). Although the apparent performance of control group seemed increasing, the actual “performance” of control group on the n-back task was always 1-back with the increasing of difficulty level. In contrast, the performance of WM training group for the n-back task was increasing from 1-back to 1 + n-back with the increasing of difficulty level. It is unnecessary and improper to compare the apparent performance of n-back task and 1-back task. **(B)** OSPAN, operation-word span task, which is an untrained WMC task as an evaluation of near transfer effect. The post-training OSPAN score for the WM training group was higher than the control group (trend-level), demonstrating higher WMC for the WM training group after training. It should be mentioned that the post-training OSPAN score was also improved after training for the control group, which might due to the familiarity effects, placebo effects of training, or easier training tasks for the control group that might improve self-efficacy.

The manipulation check of WM training was conducted first. There were no significant differences on perceived attention level, perceived difficulty level, or perceived attraction level between WM training group and control group (all *ps* > 0.373), indicating that these extra variables unlikely contributed to the differences between these two groups. More improvement, we could see from **Figure 3A** that the performance of the trained task (i.e., n-back task) of WM training group has increased overtime, and in order to demonstrate that this kind of improvement was significant, we compared the mean level (*M* = 6.719, *SD* = 1.798) achieved in the first two training sessions and the mean level (*M* = 11.688, *SD* = 3.219) achieved in the last two training sessions (according to Jaeggi et al., 2011): there was a significant improvement on the achieved difficulty level (i.e., the performance of trained task) for the WM training group, *t*(15) = -8.242, *p* < 0.001. In contrast, for the control group, the training task was always 1-back, which would not affect WMC in general as mentioned above (i.e., although the apparent performance of control group seemed increasing from **Figure 3A**, the actual “performance” of control group on the n-back task was always 1-back with the increasing of difficulty level). In summary, the profile of training indicated that participants in WM training group showed a significant improvement on a trained WMC task (i.e., n-back task) after training, but the participants in control group did not, that is, the WMC of WM training group should be higher than control group after training.

#### Near Transfer of WM Training

Near transfer refers to the effect of WM training transferring to other untrained WMC task. In the present study, the training task was n-back task (see the “Profile of WM training” above) and the near transfer task was OSPAN (see **Table 3** for the descriptive data). We used the change of OSPAN scores (i.e., post-training OSPAN scores minus pre-training OSPAN scores, the average OSPAN change was 10.063 (*SD* = 8.181) for WM training group, and 5.031 (*SD* = 7.288) for control group) as dependent valuable and conducted a one-way ANOVA (Training Group as between-participants factor: WM training vs. control). Results indicated that there was a trend-level difference between WM training group and control group for the improvement of OSPAN scores after training, *F*(1,30) = 3.374, *p* = 0.076, $\eta_{p}^{2}$ = 0.101, demonstrating that participants in WM training group showed better performance on untrained WMC task (i.e., OSPAN) after WM training compared with control group (i.e., a near transfer effect, see **Figure 3B**). Combined this result with the “Profile of the WM training” above, we inferred that WMC was improved for the WM training group after WM training, which supported H2-1. Now we have successfully manipulated the WMC, and we could examine the effect of SA and WMC on attentional control again.

**Table 3:** Working memory capacity and attentional control in Experiment 2 (means, with standard deviations in parentheses).

Indicators	Pre-training				Post-training			
	WM training		Control		WM training		Control	
	Low-SA	High-SA	Low-SA	High-SA	Low-SA	High-SA	Low-SA	High-SA
**WMC**					
OSPANs	14.656 (5.036)		14.938 (5.147)		24.719 (11.312)		19.969 (9.177)	
**Attentional control**					
Latency	359.179 (33.018)	379.813 (45.241)	369.916 (52.576)	387.399 (51.719)	333.286 (32.058)	338.738 (30.633)	382.246 (58.466)	370.708 (49.867)
Error rate	0.168 (0.101)	0.200 (0.095)	0.191 (0.138)	0.271 (0.114)	0.174 (0.115)	0.200 (0.164)	0.171 (0.131)	0.205 (0.124)

*WMC, working memory capacity; SA, state anxiety; WM training, the working memory training group; Control, the control group; OSPANs, operation-word span task scores; Latency, the latency of first correct saccade; Error rate, the percentage of incorrect saccades*.

#### Re-examination for the Effects of SA and WMC on Attentional Control

The manipulation check of SA was conducted first: the heart rate, skin conductance and MRF-3 scores were all significantly increased in the high-SA condition (all *ps* < 0.002 for both pre- and post-training), which implied that SA manipulation was successful for both pre- and post-training. We used the change of latency [i.e., post-training latency minus pre-training latency. The average change of latency under low-SA condition was -25.894 (*SD* = 20.702) for WM training group, and 12.330 (*SD* = 47.953) for control group, whereas under high-SA condition was -41.075 (*SD* = 36.636) for WM training group, and -16.691 (*SD* = 33.313) for control group] and the change of error rate [i.e., post-training error rate minus pre-training error rate. The average change of error rate under low-SA condition was 0.006 (*SD* = 0.087) for WM training group, and -0.020 (*SD* = 0.125) for control group, whereas under high-SA condition was 0.001 (*SD* = 0.132) for WM training group, and -0.067 (*SD* = 0.079) for control group] as dependent valuables. We originally would like to conduct 2 (Training Group as between-participants factor: WM training vs. control) × 2 (SA Condition as within-participants factor: low vs. high) ANCOVA for the change of latency and error rate, respectively, and the covariant variable was TA measured by T-AI. But the preliminary analysis showed that TA was not a applicable covariant variable^2^, so typical 2 × 2 ANOVA was then conducted for the change of latency and error rate respectively, regardless of TA (see **Table 3** for the descriptive data). It should be noted that there was no significant difference on T-AI between WM training and control group, *t*(30) = -1.404, *p* = 0.171, demonstrating that that TA is not the main contributor of the effects for attentional control.

**FIGURE 4:**
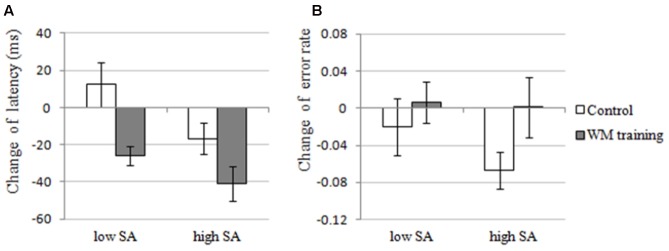
The changes of attentional control of the WM training group and the control group under low and high SA in Experiment 2. **(A)** Latency, the latency of first correct saccade, which is an indicator of attentional control, reflects the deficit of attentional control if participant need longer latency, and the change of latency was calculated by post-training latency minus pre-training latency. In Experiment 2, the main effects of Training Group and SA Condition were significant, but the interaction effect was not significant for the change of latency. **(B)** Error rate, the percentage of incorrect saccades, which is also an indicator of attentional control, reflects the deficit of attentional control if a participant performed more incorrect saccades, and the change of error rate was calculated by post-training error rate minus pre-training error rate. In Experiment 2, none of the effects were significant for the change of error rate.

The results of 2 × 2 ANOVA showed that: for the change of latency, (a) the main effect of Training Group was significant, *F*(1,30) = 7.012, *p* = 0.013, $\eta_{p}^{2}$ = 0.189, that is, the latency of WM training group decreased more than control group after training (see **Figure 4A**), demonstrating that WM training group have better attentional control than control group after training, which supported H2-2. (b) The main effect of SA condition was significant, *F*(1,30) = 22.082, *p* < 0.001, $\eta_{p}^{2}$ = 0.424, that is, the latency under high-SA condition decreased more than under low-SA condition after training (see **Figure 4A**), demonstrating that SA favors attentional control after training, which was contrary to the H2-3, and also inconsistent with the results of Experiment 1 (i.e., H1-1). (c) The Training Group × SA Condition interaction was not significant, *F*(1,29) = 2.165, *p* = 0.152, $\eta_{p}^{2}$ = 0.067, which was consistent with the results of Experiment 1. Furthermore, for the change of error rate (see **Figure 4B**), none of these effects were significant (all *ps* > 0.162).

#### Far Transfer of WM Training

Far transfer refers to the effect of WM training transferring to other untrained related tasks but not WMC tasks (e.g., attentional control tasks, fluid intelligence tasks, etc). In the present study, the training task was n-back task (see the “Profile of WM training” above), the near transfer task was OSPAN (see the “Near transfer of WM training” above), and the far transfer task was antisaccade task. As described above, the performance of WM training group on the trained task (i.e., n-back task) and untrained task (i.e., OSPAN) were improved after training. More important, WM training group have better attentional control than control group after training, which supported H2-2 and also implied that the benefit of WM training has transferred to the untrained, non-WMC task (i.e., far transfer effect).

### Discussion

In Experiment 2, we sought to examine the effect of SA and WMC on attentional control again by manipulating both SA (the same manipulation with Experiment 1) and WMC (using adaptive n-back training). Results revealed that the performance of n-back task (the trained WMC task) had been enhanced after training (see **Figure 3A**, see also Dahlin et al., 2008; Schmiedek et al., 2010; Jaeggi et al., 2011), and the performance of OSPAN (an untrained WMC task) had also been enhanced after training, demonstrating a near transfer effect of WM training (H1-1 was supported, see **Figure 3B**, see also many other WM training studies mentioned in Section “Introduction”). So we claim the manipulation of WMC was successful. More important, the latency of WM training group (the individuals who have improved WMC) in antisaccade task was decreased after training (supported H2-2, see **Figure 4A**), demonstrating that improved WMC closely related with better attentional control, which is consistent with the results of Experiment 1 (i.e., high-WMC individuals have better attentional control), and this might be a more direct evidence than previous studies that did not manipulate WMC directly. Strangely, the results also showed that SA favors attentional control (did not support H2-3, see also Booth and Sharma, 2009), which seems inconsistent with the results of Experiment 1 (we will try to explain it fully in Section “General Discussion”). And the SA × Training Group interaction (i.e., compared to the SA × WMC interaction in Experiment 1) was not significant, which is consistent with the speculation in Experiment 1: the effects of SA and WMC on attentional control are independent with each other (this will be discussed in Section “General Discussion,” too).

Besides, results of Experiment 2 also demonstrating a far transfer effect of WM training, and it might be a considerable evidence for the debate of far transfer effect mentioned in Section “Introduction.” Because (a) we used OSPAN, which is one of the most representative WMC tasks according to Engle et al. (1999), as a measurement of WMC. OSPAN is the most commonly used task to evaluate WMC in studies focused on controlled attention view of WMC, but we have not seen it was used in studies of WM training, so OSPAN here is a reliable measurement in general and also a novel measurement for WM training study. (b) Studies concerning WM training and attentional control relied heavily on the Stroop task to evaluate attentional control (Shipstead et al., 2012), Stroop task confounds attentional control and task performance, which is not a good measurement of attentional control, so the antisaccade task might be a better measurement of attentional control. Future studies should also employ different measurements of attentional control to provide different perspective of far transfer on attentional control.

However, the results of Experiment 2 must be concluded carefully given that the near transfer effect was trend-level (*p* = 0.076), which might be attributed to the unexpected enhancement of OSPAN score for control group (see **Figure 3B**). We suppose that was the reason of (a) familiarity effects, (b) placebo effects of training, and (c) the training task of control group: it might be easier than the training task of the WM training group (see **Figure 3A**, the apparent performance of control group increased faster than WM training group), leading to higher self efficacy, which might affect the performance of OSPAN. Besides, we did not found the correlation between amount of improvement on the trained task, and the amount of improvement on the near-transfer OSPAN task. That might be due to (a) the number of participant is too small (considering that WM training is very tough, we preferred to have a smaller sample size to ensure the effectiveness of training). For the WM training group, there is 16 participants, which might be insufficient for a correlation test, and (b) the individual difference of training sensitivity. Overall, all participants in WM training group performed better on OSPAN after training compared with before training, the training effect might be better for some participants who performed worse on training task (even they still performed bad after all training session).

## General Discussion

The main purposes of present study were to examine the effects of SA and WMC on attentional control using two experiments. We manipulated SA and divided participants into low- and high-WMC group in Experiment 1, and we manipulate both SA and WMC in Experiment 2. Results shows consistent positive effects of WMC on attentional control (i.e., higher WMC means better attentional control in Experiments 1 and 2), inconsistent effects of SA on attentional control (i.e., SA impaired attentional control in Experiment 1 and favored attentional control in Experiment 2), and a consistent null-effect of SA × WMC on attentional control (i.e., the possible interaction effect was not found). Here we attempted to explain these results in detail.

First, the consistent positive effect of WMC on attentional control replicated most of the previous studies about the controlled attention view of WMC. The unique contribution of present study is that we manipulated WMC directly rather than just divided participants into low- and high-WMC group, which provided more direct evidence to the controlled attention view of WMC. The exact mechanism of WMC favors attentional control is still unclear, attentional control and WMC might be the different representation of same psychological variable (or attentional control is part of the function of WMC), because WMC and attentional control have similar neural basis (i.e., prefrontal cortex) (Kane and Engle, 2002; van Veen and Carter, 2006), and ERP study showed that after WM training, the amplitudes of P300 and N160 increased significantly whereas that of P200 decreased (Zhao et al., 2013). The increase of P300 and N160 respectively implied stronger ability of updating (Gevins and Smith, 2000) and stronger concentration of task-relevant information (Mcevoy et al., 2001), and the decrease of P200 implied stronger inhibition of task-irrelevant information (Mcevoy et al., 2001). These effects above are highly correlated with attentional control, but this study is about WM training. Future research could attempt to divide attentional control and WMC in a physiological way. Besides, we manipulated WMC by WM training, which also provide new evidence of far transfer effect on attentional control, but the effectiveness of far transfer effect still needs more evidences from different attentional control indicators due to that there are also many negative evidences (e.g., Shipstead et al., 2012; Melby-Lervåg and Hulme, 2013; Melby-Lervåg et al., 2016).

Second, the inconsistent effects of SA on attentional control reflected similar inconsistent results as previous studies (see Introduction). We claim that different SA levels would lead to different effects of SA on attentional control: relatively high SA would impair attentional control, whereas relatively low or moderate SA would have little effect (or even benefit) on attentional control, because SA also has some positive effects such as enhancing arousal level and motivation, which might counteract the negative effects of SA on attentional control (sometimes the positive effects of SA might stronger than negative effects). This inference could explain the inconsistent results of Experiments 1 and 2: relatively high SA impaired attentional control in Experiment 1, whereas relatively low or moderate SA favored attentional control in Experiment 2. Evidences that support this inference are (a) the brief interview after post-training test revealed that all participants felt more relaxed in post-training test than in pre-training test, because they were more familiar and confident in completing the antisaccade in post-training test; (b) More important, we compared the pre- and post-training SA manipulation data, the heart rate and MRF-3 score in post-training test were significantly lower than in pre-training test (all *ps* < 0.003). So, the inconsistent results of SA on attentional control could be explained by our novel inference, which is also the contribution of present study. Future studies should pay more attention to induce relatively high SA when exploring similar topics. A standard multi-sources SA condition should be proposed so that we could induce relatively high SA and easily compare results of different studies.

Third, we claim that the consistent null-effect of SA × WMC on attentional control implied that the effects of SA and WMC might be independent with each other, that is, SA and WMC might affect different aspects of attentional control. We think that SA will affect stimulus-driven system, whereas WMC will affect goal-directed system. The evidence for SA affects stimulus-driven system is that Pacheco-Unguetti et al. (2010) had explored the effects of TA and SA on attentional network (orienting, alerting, and executive control), they found that TA impaired executive control (which is more like goal-directed system), whereas SA was associated with an over-functioning of the alerting and orienting (which are more like stimulus-driven system). As for the evidence for WMC affects goal-directed system is that high-WMC individuals perform better top–down attentional control such as they were better at resisting distractors (Kane et al., 2001; Unsworth et al., 2004; Unsworth and Spillers, 2010), they could amplify task-relevant information or inhibit task-irrelevant information according to task requirement (Colflesh and Conway, 2007), and they searched object (top–down) by keeping the features in their minds (Bleckley et al., 2014). Besides, the neural basis of WMC is prefrontal cortex, which is also the basis of top–down attentional control (Kane and Engle, 2002; van Veen and Carter, 2006). Future research could consider exploring this speculation about the relationship between SA and WMC.

It should be highlighted that the relationship between anxiety and attentional control predicted by Attentional Control Theory needs to be refined, because anxiety could be divided into TA and SA and the effects of SA on attentional control are complex as mentioned above. According to Pacheco-Unguetti et al. (2010), TA impairs attentional control through impairing goal-directed system, and SA impairs attentional control by favoring stimulus-driven system. This explanation is also helpful for understanding the null-effect of the SA × WMC interaction (e.g., the present study, see also Edwards M.S. et al., 2015; Wood et al., 2015). SA and WMC affect different aspects of attentional control, so it is more difficult to observe this interaction compared with TA × WMC interaction (TA and WMC affect the same aspects of attentional control, e.g., Johnson and Gronlund, 2009; Wright et al., 2014). Another refinement should be considered is the SA level, that is, relatively high SA level might be necessary for observing the negative effect of SA on attentional control (already discussed above).

One shortcoming of present study is that the effects on antisaccade error rate are mostly null-effect in present study (except for the SA on error rate in Experiment 1). High-WMC individuals did not show lower error rate in Experiments 1 and 2, and SA had little effect on error rate in Experiment 2. A similar pattern was reported by Derakshan et al. (2009): they argued that error rate is more suitable to become an evaluation of antisaccade task performance rather than attentional control. It seems that error rate is probably not a sensitive enough indicator of attentional control in antisaccade task. Future studies could consider regarding error rate as a task performance indicator rather than an attentional control indicator. Besides, given that the education levels in the present study were inconsistent (i.e., we included both undergraduate and graduate students, and people who have higher education level might imply higher ability, higher WMC or attentional control), future studies could pay more attention to the education levels of participants in order to provide more reliable evidence and extend the result to people with different education levels.

Taken in sum, the present study implies a complex relationship between SA and attentional control, emphasizes the important promotion of WMC on attentional control, and denies the possible interaction of SA and WMC. In detail: (a) we found that the effect of SA on attentional control will depend on the SA level, that is, relatively high SA level might be necessary for observing the negative effect of SA on attentional control; (b) we manipulated WMC directly by WM training, and provided more reliable evidence for the importance of high-WMC on better attentional control and a new supportive evidence on far transfer effect of WM training; (c) we did not found the interaction of SA and WMC, and we speculated that the effects of SA and WMC on attentional control might be independent with each other, that is, SA and WMC might affect different aspects of attentional control (e.g., SA will affect stimulus-driven system, whereas WMC will affect goal-directed system).

## Ethics Statement

The present study was approved by Beijing Sport University Institutional Review Board (BSUIRB) (Approval Number: 2015037). Before experiment, all participants provided informed consent to confirm that they were clear about the details and possible uncomfortable feelings in the experiment. Only when participants agreed can we start experiment. Participants could drop out at anytime during the experiment, and we also have debriefing period after experiment. Vulnerable populations were not involved in the present study.

## Author Contributions

XL and LZ contributed to the conception and design of the work. XL contributed to the acquisition of data. XL and LZ contributed to the analysis and interpretation of data for the work. XL, LZ, and JW wrote and revised the manuscript. XL, LZ, and JW approve the final version of the manuscript. XL, LZ, and JW agrees to be accountable for all aspects of the work in ensuring that questions related to the accuracy or integrity of any part of the work are appropriately investigated and resolved.

## Conflict of Interest Statement

The authors declare that the research was conducted in the absence of any commercial or financial relationships that could be construed as a potential conflict of interest.The reviewer YW and handling Editor declared their shared affiliation and the handling Editor states that the process nevertheless met the standards of a fair and objective review.

## Footnotes

1 An applicable covariant variable should meet two prerequisites at the same time: (a) correlate with dependent variable and (b) independent with independent variable. Here the preliminary analysis of ANCOVA revealed that TA was not applicable as a covariant variable, because the main effect of TA was not significant, that is, the covariant variable was unrelated with dependent variables, for latency: *F*(1,52) = 0.771, *p* = 0.384, for error rate: *F*(1,52) = 1.703, *p* = 0.198, which did not meet the first prerequisite, so we exclude TA and conducted typical ANOVA.

2 The reason to include TA as covariant variable here is that there might be differences on TA between WM training group and control group, which might affect attentional control but we don’t concern it. Here the preliminary analysis of ANCOVA revealed that TA was not applicable as a covariant variable, because the main effect of TA was not significant, that is, the covariant variable was unrelated with dependent variables, for the change of latency: *F*(1,28) = 0.946, *p* = 0.339, for the change of error rate: *F*(1,28) = 0.011, *p* = 0.918, which did not meet the first prerequisite of ANCOVA, so we exclude TA and conducted typical ANOVA.

## Abbreviations

OSPAN: operation-word span task
SA: state anxiety
TA: trait anxiety
WM training: working memory training
WMC: working memory capacity.
